# The CpxAR Two-Component System Contributes to Growth, Stress Resistance, and Virulence of *Actinobacillus pleuropneumoniae* by Upregulating *wecA* Transcription

**DOI:** 10.3389/fmicb.2020.01026

**Published:** 2020-05-21

**Authors:** Kang Yan, Ting Liu, Benzhen Duan, Feng Liu, Manman Cao, Wei Peng, Qi Dai, Huanchun Chen, Fangyan Yuan, Weicheng Bei

**Affiliations:** ^1^State Key Laboratory of Agricultural Microbiology, College of Veterinary Medicine, Huazhong Agricultural University, Wuhan, China; ^2^State Key Laboratory of Genetically Engineered Veterinary Vaccines, Qingdao, China; ^3^Key Laboratory of Prevention and Control Agents for Animal Bacteriosis (Ministry of Agriculture), Institute of Animal Husbandry and Veterinary Sciences, Hubei Academy of Agricultural Sciences, Wuhan, China; ^4^The Cooperative Innovation Center for Sustainable Pig Production, Huazhong Agricultural University, Wuhan, China

**Keywords:** CpxAR, Growth, Stress resistance, Virulence, WecA

## Abstract

*Actinobacillus pleuropneumoniae* is the pathogen of porcine contagious pleuropneumonia. In *A. pleuropneumoniae*, the CpxAR two-component system is essential for fitness and growth. The O-antigen protrudes from the outer membrane to the exterior of the cell, and the outer membrane serves as a barrier that helps the bacteria to survive in harsh environments. WecA, a undecaprenyl phosphate GlcNAc-1-phosphate transferase, is involved in O-antigen repeating unit biosynthesis. In this study, we investigated the role of CpxAR in the expression of *wecA* in *A. pleuropneumoniae*. Our results revealed that CpxR positively regulates *wecA* expression by directly binding to the putative promoter region of *wecA*. Wild-type, Δ*cpxAR*, Δ*wecA*, and complemented strains were investigated under serum, oxidative, and osmotic stresses. The Δ*cpxAR* and Δ*wecA* strains were more susceptible to these stresses than the wild-type, but the complemented strains showed phenotypes similar to those of the wild-type. Mice infected with the Δ*cpxAR* and Δ*wecA* strains exhibited lower mortality and bacterial loads in the lung than those infected with the wild-type or complemented strains. This study reveals that the CpxAR two-component system contributes to *A. pleuropneumoniae* growth, stress resistance, and virulence, by upregulating expression of *wecA.* Our findings provide new insight into the pathogenesis of *A. pleuropneumoniae*.

## Introduction

Porcine contagious pleuropneumonia is a highly contagious respiratory disease that causes considerable losses to the global swine industry (Sassu et al., 2018). Its causative agent, *Actinobacillus pleuropneumoniae*, is a Gram-negative bacterium that can be differentiated into 18 serovars (Bosse et al., 2018a, b). *A. pleuropneumoniae* is transmitted by direct contact or aerosol and can infect swine of all ages (Sheehan et al., 2003). Several virulence factors are involved in the pathogenesis of *A. pleuropneumoniae*, including capsule polysaccharide, lipopolysaccharide, proteases, Apx toxins, outer membrane proteins, and transferrin-binding proteins (Chiers et al., 2010).

Two-component systems (TCSs) are one of the major strategies of signal transduction used by bacteria to sense and respond to their changing environment (Jacob-Dubuisson et al., 2018). The Cpx TCS consists of the sensor kinase CpxA and the response regulator CpxR. CpxR can regulate the transcription of downstream genes after CpxA transfers a phosphate group to CpxR (Raivio and Silhavy, 1997; Vogt and Raivio, 2012). Previous studies have reported that the CpxAR TCS enables bacteria to respond to envelope stress and environmental stresses such as oxidative and osmotic stress (Bury-Mone et al., 2009; Srinivasan et al., 2012; Bontemps-Gallo et al., 2015; Cao et al., 2018; Lopez et al., 2018; Matter et al., 2018).

Gram-negative bacteria are surrounded by an outer membrane (OM). The OM serves as a barrier that helps them to survive in harsh environments (Nikaido, 2003). Lipopolysaccharide (LPS) is the central constituent of the OM, playing a vital role in interactions with the environment, including host organisms of pathogens. LPS is composed of O-antigen, lipid A, and core oligosaccharides (Raetz and Whitfield, 2002). The O-antigen is distal to the OM (Raetz and Whitfield, 2002). In *Salmonella enterica* serovar Typhimurium, the O-antigen helps cells to evade the complement cascade (Murray et al., 2006). In *Erwinia amylovora*, *Bradyrhizobium japonicum*, and *Escherichia coli*, the O-antigen protects cells against oxidative stress (Berry et al., 2009; Noh et al., 2015; Zheng et al., 2019). O-antigen was reported to protect *B. japonicum* and *S. enterica* serovar Typhi against osmotic stress (Bittner et al., 2002; Noh et al., 2015). In addition, some stress conditions influence O-antigen expression (Bittner et al., 2002; Silva-Valenzuela et al., 2016). In general, the genes involved in O-antigen biosynthesis are found in O-antigen gene clusters and close to each other (Skurnik et al., 1999; Yi et al., 2005; Liu et al., 2008, 2019). WecA (Rfe) initiates O-antigen repeating unit biosynthesis (Alexander and Valvano, 1994; Rick et al., 1994; Clarke et al., 1995; Lehrer et al., 2007).

Previous studies showed that the Cpx TCS is related to mediation of host environmental fitness (Ruiz and Silhavy, 2005; Yamamoto and Ishihama, 2006; Vogt and Raivio, 2012; Delhaye et al., 2016; Pezza et al., 2016), that the O-antigen helps bacteria to survive in harsh environments (Bittner et al., 2002; Noh et al., 2015; Silva-Valenzuela et al., 2016; Zheng et al., 2019), and that the CpxAR TCS is activated by overexpression of O-antigen (Bengoechea et al., 2002). However, it remained unclear whether the CpxAR system regulates O-antigen biosynthesis and affects stress resistance in *A. pleuropneumoniae*. This study reveals that CpxR can directly bind to the putative promoter region of *wecA* and upregulate *wecA* gene expression in *A. pleuropneumoniae*. Δ*wecA* and Δ*cpxAR* strains exhibited slower growth and were more sensitive to stressful conditions, including serum, oxidative, and osmotic stresses, than the wild-type strain and complemented mutants. Furthermore, *in vivo* evidence from a mouse infection model demonstrated that virulence of the Δ*cpxAR* and Δ*wecA* strains was attenuated compared with that of the wild-type. Our findings provide insight into the CpxAR system and WecA function in *A. pleuropneumoniae*.

## Materials and Methods

### Bacterial Strains and Culture Conditions

The bacterial strains, primers, and proteins used in this study are described in Table 1. Tryptic soy broth (TSB; Difco Laboratories, Detroit, MI, United States) containing 10 μg mL^–1^ nicotinamide adenine dinucleotide (NAD) and 10% (v/v) newborn bovine serum was used to grow *A. pleuropneumoniae* strains, and the solid medium was tryptic soy agar (TSA; Difco Laboratories). For antibiotic selection, 5 μg mL^–1^ chloramphenicol were added to the medium when necessary. *E. coli* strains were grown in Luria-Bertani broth or agar; appropriate antibiotics or 50 μg mL^–1^ diaminopimelic acid (Sigma-Aldrich, St. Louis, United States) were added when needed. Wild-type *A. pleuropneumoniae* in this study was strain 4074.

**TABLE 1:** Bacterial strains, plasmids, and proteins used in this study.

Strains, plasmids, and proteins	Characteristics	Source or reference
Strains		
*A. pleuropneumoniae*		
4074	*A. pleuropneumoniae* reference strain of serovar 1; wild-type strain	Laboratory
Δ*cpxAR*	*A. pleuropneumoniae* 4074 *cpxAR*-deletion mutant	Laboratory
CΔ*cpxAR*	Complemented strain of Δ*cpxAR*; Cm^r^	Laboratory
E. coli		
*DH5 a*	Cloning host	Laboratory
*β2155*	Transconjugation donor for constructing mutant strain	From Prof. Gerald-F. Gerlach
Plasmids		
pEMOC2	Transconjugation vector: ColE1 ori mob RP4 sacB, AmprCm^r^	From Prof. Gerald-F. Gerlach
pEΔ*wecA*	Up- and down-stream arms of *wecA* were ligated sequentially into pEMOC2, and used as the transconjugation vector for *wecA* gene deletion	This study
pJFF224-XN	*E. coli*-APP shuttle vector: RSF1010 replicon; mob oriV, Cm^r^	Laboratory
pJFF-*wecA*	pJFF224-XN carrying the intact *wecA*	This study
Proteins		
rCpxR	For EMSA assay	Laboratory

*Cm^r^, Chloramphenicol resistance.*

The mutant strain Δ*wecA* and the complemented strain CΔ*wecA* were constructed as described previously (Frey, 1992; Li et al., 2018). Polymerase chain reaction and sequencing (data not shown) were used to verify the mutant strain and the complemented strain. Briefly, the upstream and downstream segments of *wecA* were amplified. These segments were combined by overlap PCR. Primers used in the PCR are listed in Table 2. The overlapped product was ligated into pEMOC2 to generate pEΔ*wecA*. pEΔ*wecA* was used to construct a Δ*wecA* mutant by double crossover recombination. The intact *wecA* gene was amplified and cloned into pJFF224-XN to obtain pJFF-*wecA*, which was electroporated into the Δ*wecA* strain. The resultant strain, CΔ*wecA*, was selected on TSA containing chloramphenicol, bovine serum, and NAD.

**TABLE 2:** Primers used in this study.

Primers	Sequence (5’–3’)	Source or reference
**For mutant construction**		
*wecA*-up-F/R	CTGTCGACAACAATGACCATACCGAGTAATAAGC TTTGTGGTGTCGTTCGCCAGTCTGAGTCGTATGAA ACGCAGAGCGAAAAG	This study
*wecA*-down-F/R	CTTTTCGCTCTGCGTTTCATACGACTCAGACTGGC GAACGACACCACAAA ATGCGGCCGCCCTAATGCGACACGGAAACC	This study
*wecA*-extrrior-F/R	ACCGATTTCCGGCGCAAGAG TGGCGAAGCAAAGAACAGTAATAGAG	This study
*wecA*-interior-F/R	TGCCCTTGACCTTGGGTGCTA TCTTGCGGCGGTGACTATTCTA	This study
**For complement construction**		
*wecA*-F/R	GCGTCGACATGTGGCTTACTTTTCTTGCTGTTT TAGCGGCCGCTTACTCACTACCGATTTTGAGCTTTCTTTTC	This study
**For qRT-PCR**		
16S rRNA-F/R	CCATGCCGCGTGAATGA TTCCTCGCTACCGAAAGAACTT	Subashchandrabose et al., 2013
WecA-F/R	ATACGCCGATGGTAGAGCAA ATCGATATGGTGGCGGTCAT	This study
**For emsa assay**		
WecA-E-F/R	CTGAATAGCGTTGATCGACAAGGTT	This study
	GGGGTTAGCTCCCTCAAAATCAATA	

### RNA Extraction and Reverse Transcription Quantitative-PCR

The wild-type strain and Δ*cpxAR* were grown in TSB (containing inactivated bovine serum and NAD) overnight, and then diluted 1:100 into fresh medium and grown to an OD_600 *nm*_ of 0.6. Bacteria were harvested and treated with serum, 1.5 M NaCl, or 0.5 mM H_2_O_2_. RNA was extracted using a Bacteria Total RNA Isolation Kit (Sangon Biotech, China), then used for cDNA synthesis using HiScript II Q RT SuperMix for qRT-PCR (+gDNA wiper) (Vazyme, China). Quantitative PCR was performed by a one-step reaction in a ViiA^TM^ 7 real-time PCR system (Walters et al., 2006). The reaction mixtures were prepared according to the manufacturer’s instructions for SYBR Green Master Mix (Vazyme). The 16S rRNA gene was used as an endogenous control. Primers used in the qRT-PCR analysis are listed in Table 2. The 2^–^*^ΔCt^* method was used to quantify and compare gene expression (Pfaffl, 2001). To determine whether *wecA*, *APPSER1_RS08540*, *wecB*, *wecC*, *rffC*, *rffA*, and *wzxE* lie in one operon, cDNA from the wild-type strain and intergenic region-spanning primers (Supplementary Table S1) were used for PCR analysis.

### Electrophoretic Mobility Shift Assays (EMSAs)

A DNA probe containing the putative *wecA* promoter region (272 bp) was amplified from the wild-type strain by PCR with primers WecA-E-F/R (Table 2). A mutant probe for the putative *wecA* promoter region (with TTTAC in the putative CpxR-P binding box deleted, 267 bp) was synthesized, and was used to explore the specificity of CpxR-P binding to the putative *wecA* promoter region. These two DNA probes were labeled by using an EMSA Probe Biotin Labeling Kit (Beyotime, China). Recombinant CpxR protein was phosphorylated by acetyl phosphate (Sigma, United States) (Pogliano et al., 1997). EMSAs were performed as previously described with some modifications (Li et al., 2018).

### Growth Characteristics of Strains

Overnight bacterial cultures of *A. pleuropneumoniae* 4074, Δ*cpxAR*, Δ*wecA*, CΔ*cpxAR*, and CΔ*wecA* were diluted 1:1000 in fresh medium, and were grown at 37°C. OD_600_ was measured using an Eppendorf Biophotometer (Eppendorf, Hamburg, Germany) every hour. When OD_600_ of the cultures reached 0.3, 0.6, 1.5, and 2.5, respectively, cultures were serially diluted tenfold in phosphate-buffered saline (PBS) and plated on TSA.

### Serum Bactericidal Assays

Swine serum was collected from healthy piglets with no history of *A. pleuropneumoniae* infection and no clinical signs. All the collected sera were determined to be *A. pleuropneumoniae*-negative by ApxIV-enzyme-linked immunosorbent assays (Kqbio, China). Some sera were heat-inactivated at 56°C for 30 min. The wild-type strain, Δ*cpxAR*, Δ*wecA*, CΔ*cpxAR*, and CΔ*wecA* were grown to mid-log phase. Bacteria were collected and washed twice with PBS. Bacterial suspensions (50 μL) were mixed with 450 μL fresh serum or heat-inactivated serum. After 1 h of incubation at 37°C, samples were diluted serially and plated on TSA (containing bovine serum and NAD) for enumeration of colony-forming units (CFU). The percentage bacterial survival was calculated by comparing the number of colonies that survived in fresh serum to the number that survived in heat-treated serum.

### Oxidative and Osmotic Stress Resistance Assays

Oxidative stress assays were performed as previously described with some modifications (Li et al., 2019). Osmotic stress assays were carried out at 1.5 M NaCl (Li et al., 2004; Linnes et al., 2013). Overnight cultures were diluted 1:100 in TSB containing 10% inactivated bovine serum and 10 μg/mL NAD, and the bacterial cultures were grown in TSB to mid-logarithmic phase. Bacteria were harvested by centrifugation, resuspended in TSB containing 0.5 mM H_2_O_2_ (for oxidative stress) or 1.5 M NaCl (for osmotic stress), and incubated for 30 min. Control samples were cultivated in TSB without any addition. After incubation, the samples were serially diluted, plated on TSA containing inactivated bovine serum and NAD, and incubated at 37°C for 12 h. Survival rate was calculated by dividing the number of CFU in the stressed sample by that in the control sample.

### Bacterial Virulence *in vivo*

Female BALB/c mice (6-weeks-old) were purchased from the Center for Disease Control of Hubei Province (Hubei CDC, Wuhan, China). All animal experiments were performed in accordance with the guidelines of the Laboratory Animal Monitoring Committee of Huazhong Agricultural University.

The infection assay was performed as described previously with some modifications (Li et al., 2018). Mice were randomly divided into five groups (8 per group): wild-type, Δ*cpxAR*, Δ*wecA*, CΔ*cpxAR*, and CΔ*wecA*-treated. Briefly, overnight bacterial cultures were diluted 1:100 in fresh medium and then incubated again until an OD_600_ of 0.6 was reached. Bacteria were collected and washed twice with PBS. Each group of mice was injected via the abdominal cavity with PBS containing 1 × 10^7^ CFU. Survival time was monitored for 5 days post-infection. At day five post-infection, surviving mice were euthanized for postmortem examination. When infected mice died, or were humanely sacrificed, their lungs were fixed in 10% neutral-buffered formalin. Thin sections were stained with hematoxylin/eosin.

Bacterial burden experiments were performed as previously described with some modifications (Li et al., 2018). Female BALB/c mice (six per group) were challenged intraperitoneally with 2 × 10^6^ CFU of each strain. Bacteria in the lung were counted at hour six post-infection.

### Statistical Analysis

Statistical analysis was conducted using GraphPad Prism software (San Diego, United States). The data obtained from the present study are expressed as mean ± SD. Student’s *t*-test was used to analyze differences between groups. The log-rank test was performed to present differences in survival rate. Statistically significant differences are presented at the level *P* < 0.05 and *P* < 0.01.

## Results

### Expression of *wecA* is Upregulated in *A. pleuropneumoniae* Under Environmental Stresses

**FIGURE 1:**
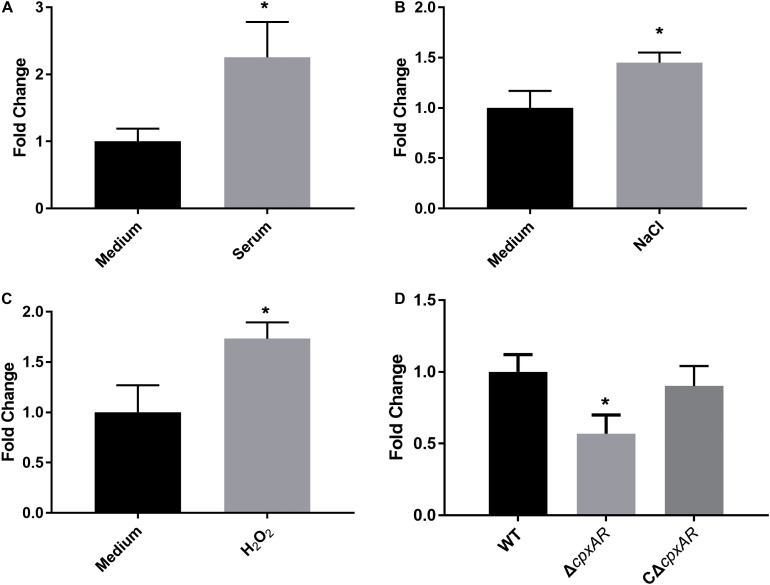
Upregulation of transcription level of *wecA* after exposure of wild-type *Actinobacillus pleuropneumoniae* (strain 4074) to serum **(A)**, NaCl **(B)** and H_2_O_2_ **(C)**. Quantitative real-time PCR analysis of expression of *wecA* in wild-type and Δ*cpxAR* strains **(D)**,^∗^*p* < 0.05.

To investigate whether WecA is involved in stress resistance, the wild-type strain *A. pleuropneumoniae* 4074 was treated with serum, NaCl, and H_2_O_2_, respectively. The transcription level of *wecA* was upregulated after exposure to serum, NaCl and H_2_O_2_ (Figure 1).

### CpxAR TCS Influences Transcription of *wecA*

To investigate the effect of the CpxAR TCS on the transcription of *wecA*, the expression of *wecA* in Δ*cpxAR* and wild-type strains was determined by qRT-PCR. As shown in Figure 1D, the expression of *wecA* in the Δ*cpxAR* mutant decreased significantly (*P* < 0.05) compared with that in the wild-type strain. Complementation of the *cpxAR* mutation restored the expression level of *wecA*.

### CpxR-P Directly Binds to the Putative *wecA* Promoter Region

**FIGURE 2:**
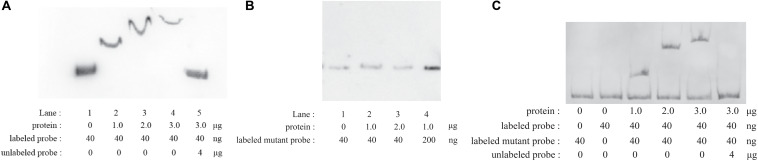
Electrophoretic mobility shift assays. Labeled *wecA* putative promoter region **(A)** or labeled mutant *wecA* putative promoter region **(B)** were incubated with various concentrations of CpxR-P. Labeled *wecA* putative promoter region and labeled mutant *wecA* putative promoter region were incubated with various concentrations of CpxR-P **(C)**.

EMSAs were performed with phosphorylated CpxR (CpxR-P) and a segment containing the putative *wecA* promoter region. CpxR-P slowed down the movement of the putative *wecA* promoter region in a dose-dependent manner, and the competition control demonstrated the specificity of CpxR-P binding (Figure 2A). CpxR-P was unable to bind to a mutant *wecA* putative promoter region in which TTTAC was deleted from the putative CpxR-P binding box (Figure 2B). The addition of mutant probe did not affect the binding of CpxR-P and the putative promoter region of *wecA* (Figure 2C). These results showed the specificity of the binding of CpxR-P and the putative *wecA* promoter region.

### Growth Characteristics of *A. pleuropneumoniae* Strains

**FIGURE 3:**
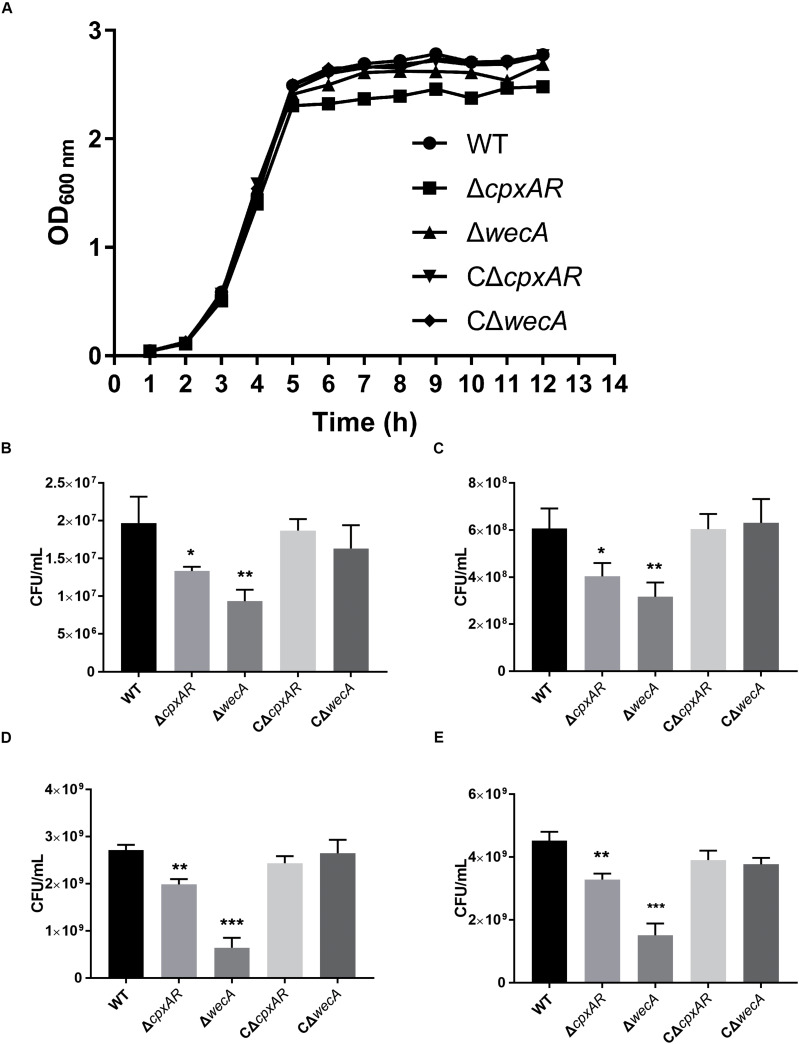
Growth characteristics of *A. pleuropneumoniae* strains. Growth curves of wild-type, Δ*cpxAR*, Δ*wecA*, CΔ*cpxAR*, and CΔ*wecA* strains **(A)**. When the OD_600_ of the bacterial cultures reached 0.3 **(B)**, 0.6 **(C)**, 1.5 **(D)**, and 2.5 **(E)**, the cultures were serially diluted and plated onto tryptic soy agar. Data are presented as the mean ± SD. Significant differences are presented at the levels ^∗^*p* < 0.05, ^∗∗^*p* < 0.01, and ^∗∗∗^*p* < 0.001.

We constructed Δ*wecA* mutant and complemented strain (Supplementary Figure S1). The growth properties of the wild-type, Δ*cpxAR*, Δ*wecA*, CΔ*cpxAR*, and CΔ*wecA A. pleuropneumoniae* strains were investigated. When strains were grown in TSB, both Δ*cpxAR* and Δ*wecA* mutants exhibited growth defects, but complementation of the strains restored the growth properties. The trends of Δ*cpxAR* and Δ*wecA* growth curves in TSB were similar to those of the wild-type strain. However, as shown in Figure 3A, in the stationary phase, OD_600_ values of Δ*cpxAR* and Δ*wecA* were lower than those of the wild-type and complemented strains. When the OD_600_ value of the bacterial cultures reached 0.3, 0.6, 1.5, and 2.5, respectively, cultures were serially diluted and plated onto TSA. The number of viable bacteria of Δ*cpxAR* and Δ*wecA* strains was lower than that of wild-type strain at each OD_600_ value, whereas no significant difference was observed in the number of viable bacteria between CΔ*wecA* or CΔ*cpxAR* and the wild-type strain (Figures 3B–E).

### Δ*cpxAR* and Δ*wecA* Strains Exhibit Reduced Resistance to Serum Killing

**FIGURE 4:**
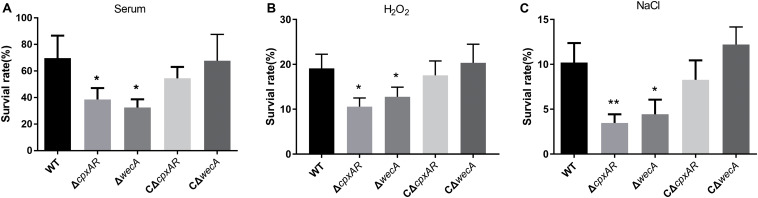
Serum and stress resistance assays. The strains were treated with serum **(A)**, H_2_O_2_ **(B)**, NaCl **(C)**, or tryptic soy broth, then bacterial survival rates were calculated. Significant differences are presented at the levels ^∗^*p* < 0.05, ^∗∗^*p* < 0.01, and ^∗∗∗^*p* < 0.001.

To examine whether the *cpxAR* and *wecA* genes are involved in serum resistance, the survival rates of wild-type, Δ*cpxAR*, Δ*wecA*, CΔ*cpxAR*, and CΔ*wecA* strains in serum were examined. The Δ*cpxAR* and Δ*wecA* mutants were significantly more sensitive to porcine serum than the wild-type, and the complemented strains were observed to have restored serum resistance (Figure 4A).

### Δ*cpxAR* and Δ*wecA* Mutants Exhibit Reduced Tolerance to H_2_O_2_ and NaCl

To investigate whether *cpxAR* and *wecA* are involved in oxidative and osmotic stress tolerance, we tested the survival rates of wild-type, Δ*cpxAR*, Δ*wecA*, CΔ*cpxAR*, and CΔ*wecA* strains when they were exposed to 0.5 mM hydrogen peroxide or 1.5 M NaCl. On treatment with 0.5 mM hydrogen peroxide, the survival rates of the *cpxAR* and *wecA* mutant strains were significantly lower than that of the wild-type (Figure 4B). When the bacteria were exposed to NaCl-induced osmotic stress, the survival rates of the Δ*cpxAR* and Δ*wecA* strains were lower than that of the wild-type (Figure 4C). Complementation of the strains restored their original phenotypes. These results suggest that CpxAR and WecA are involved in oxidative and osmotic stress tolerance.

### Δ*cpxAR* and Δ*wecA* Strains Display Attenuated Virulence and Colonization in a Mouse Model

**FIGURE 5:**
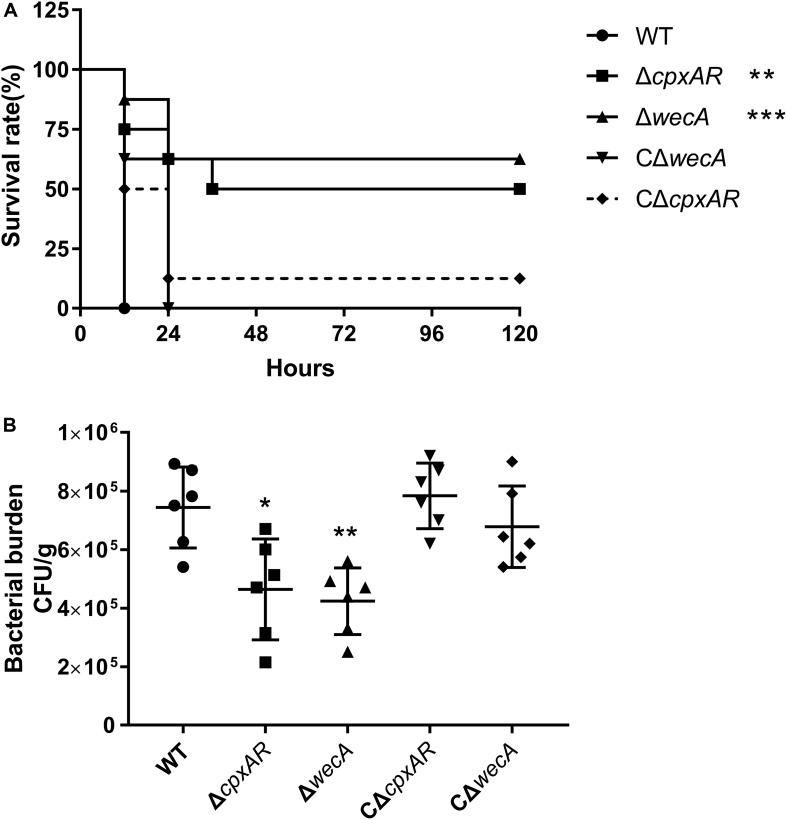
Attenuated virulence and colonization of *A. pleuropneumoniae* Δ*cpxAR* and Δ*wecA* strains in a BALB/c mouse model. **(A)** Survival curves of mice infected with *A. pleuropneumoniae* strains. Wild-type, Δ*cpxAR*, Δ*wecA*, CΔ*cpxAR*, and CΔ*wecA*-treated mice were monitored daily for 5 days post-infection. **(B)** Bacterial burden in lung. Mice were inoculated intraperitoneally with *A. pleuropneumoniae* strains. At hour six post-infection, lung samples were isolated to determine bacterial burdens. Significant differences are presented at the levels ^∗^*p* < 0.05, ^∗∗^*p* < 0.01, and ^∗∗∗^*p* < 0.001.

To explore the roles of CpxAR and WecA in the virulence of *A. pleuropneumoniae*, a BALB/c mouse model was used. As shown in Figure 5A, the survival rates of mice at 5 days were 0, 50, 62.5, 12.5, and 0% in the wild-type, Δ*cpxAR*, Δ*wecA*, CΔ*cpxAR*, and CΔ*wecA*-treated groups, respectively.

As shown in Figure 5B, a significant difference (*P* < 0.05) in the number of bacteria recovered from lung was found between the wild-type and Δ*cpxAR*-treated groups, and there was a highly significant difference (*P* < 0.01) in the number of bacteria recovered from lung between the wild-type and Δ*wecA*-treated groups.

**FIGURE 6:**
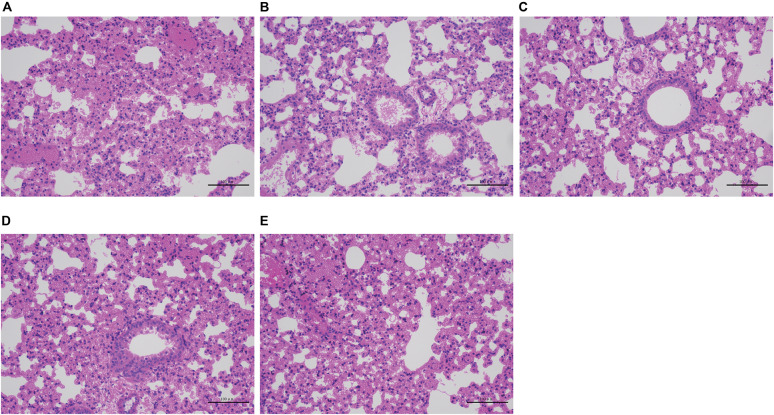
Histopathological assays. **(A–E)** Lung sections from wild-type, Δ*cpxAR*, Δ*wecA*, CΔ*cpxAR*, and CΔ*wecA*-treated mouse groups, respectively.

Severe acute hemorrhagic pneumonia symptoms were observed in mice in the wild-type, CΔ*cpxAR*, and CΔ*wecA* treatment groups, for example, thickened alveolar walls, extensive infiltration of erythrocytes, and more inflammatory cells in alveoli, whereas sections from Δc*pxAR-* and Δ*wecA*-treated mice exhibited less severe pathological changes (Figure 6). These results revealed that the CpxAR TCS and WecA contribute to *A. pleuropneumoniae* virulence.

## Discussion

In Gram-negative bacteria, the envelope has been reported to be essential for nutrient transport, adhesion, viability, stress resistance, and virulence (Bury-Mone et al., 2009). The envelope consists of the inner membrane and the OM. LPS is one of the central constituents of the OM. O-antigen, which is one constituent of LPS, is distal to the OM (Raetz and Whitfield, 2002). Previous studies have revealed that O-antigen plays a crucial role in stress tolerance and immune evasion in Gram-negative bacteria (Murray et al., 2006; Berry et al., 2009; Duerr et al., 2009; Wells et al., 2014; Noh et al., 2015; Zheng et al., 2019).

The Cpx system was identified as an important regulatory system, playing a major role in envelope stress resistance, cell wall integrity, stress tolerance, and virulence regulation in Gram-negative bacteria (Bengoechea et al., 2002; Ruiz and Silhavy, 2005; Rowley et al., 2006; Yamamoto and Ishihama, 2006; Vogt and Raivio, 2012; Raivio et al., 2013; Delhaye et al., 2016; Pezza et al., 2016). Since both O-antigen and the Cpx system are responsible for stress tolerance, it is necessary to examine their relationship in *A. pleuropneumoniae*.

Results here showed that the expression of *wecA* was upregulated after exposure of *A. pleuropneumoniae* to serum, NaCl, or H_2_O_2_. Activated CpxR enabled bacteria to adapt to the environmental stresses by regulating gene expression. The CpxR-binding consensus sequence was found to be GTAAA-(N)_4–8_-GTAAA, or TTTAC-(N)_4–8_-TTTAC (De Wulf et al., 2002; Yamamoto and Ishihama, 2006; Keilwagen et al., 2009; Srinivasan et al., 2012). Here, the putative CpxR binding sequence (TTTAC-N_7_-TTTAT) was located 46–30 bp upstream of the *wecA* start codon. Results showed that CpxR-P slowed down the movement of the putative *wecA* promoter region in EMSA, CpxR-P was unable to bind to a mutant *wecA* putative promoter region (in which TTTAC was deleted from the putative CpxR-P binding box), and the competition control demonstrated the specificity of CpxR-P binding. These results indicate that CpxR-P was able to bind to the putative CpxR-P binding box. The DNA–protein binding was not saturated. This may be because the concentration of DNA probe was too low, or the protein concentration was too high, for the DNA–protein reaction to be maximal. The promoter was predicted using Softberry^1^ (Supplementary Figure S3). As shown in Supplementary Figure S3, the CpxR-P binding site was located downstream of the putative *wecA* promoter. This finding was in accordance with another study, where the CpxR-binding site was located downstream of the *cpxP* promoter (Yamamoto and Ishihama, 2006). EMSA and qRT-PCR results revealed that CpxR-P directly bound to the putative *wecA* promoter region and positively regulated *wecA* gene expression. Previous studies revealed that the genes related to O-antigen biosynthesis were in one operon or several transcriptional units (Skurnik et al., 1999; Yi et al., 2005; Liu et al., 2008, 2019). The small intergenic space between *wecA*, *APPSER1_RS08540*, *wecB*, *wecC*, *rffC*, *rffA*, and *wzxE* in *A. pleuropneumoniae* suggested that these genes might be co-transcribed as one mRNA. Our RT-PCR results confirmed this speculation (Supplementary Figure S2). These results reveal that CpxAR is involved in O-antigen biosynthesis in *A. pleuropneumoniae*.

The CpxAR TCS upregulated the expression of genes related to O-antigen biosynthesis to resist environment stresses. Bengoechea et al. studied *Yersinia enterocolitica* and reported that overexpression of Wzz resulted in the generation of more O-antigen, thus activating the CpxA-CpxR TCS (Bengoechea et al., 2002). Both their study and our results indicate that bacteria should maintain a proper amount of O-antigen so as to be adaptable to environmental stresses.

One of our previous studies reported that the deletion of *cpxAR* from *A. pleuropneumoniae* affected growth of the bacterium (Li et al., 2018). This report was in accordance with the findings of a study in which deletion of the Cpx TCS affected the growth of *E. coli* (Delhaye et al., 2016). The present study revealed that viable bacterial numbers of Δ*cpxAR* and Δ*wecA* strains were lower than those of the wild-type strain at the OD_600_ values 0.3, 0.6, 1.5, and 2.5, but that, before the stationary phase, no significant difference in the OD_600_ values was observed between Δ*cpxAR* or Δ*wecA* and the wild-type strain (Figure 3). These two results might be due to the fact that the differences in the amount of O-antigen produced resulted in different surface structures of bacteria, thus leading to different absorbances.

The maximum number of CFU/mL for the mutants was less than that of the wild-type and complemented strains. The maximum number of CFU/mL for strain Δ*wecA* (4 × 10^9^) was approximately equal to that of the wild-type strain in the late exponential phase, which might be explained by previous reports that the production of O-antigen was associated with the growth-phase; that in *S. enterica* serovar Typhi, O-antigen increased in the late exponential and stationary phases (Rojas et al., 2001; Bittner et al., 2004); and that bacteria modify LPS structures, promoting survival in various growth conditions (Bengoechea et al., 2003; Whitfield and Trent, 2014). Our study results indicated that CpxAR affected cell growth by regulating the expression of *wecA*.

Bacterial resistance to serum is vital for pathogenicity. Previous studies reported that in *S. enterica* serovar Typhimurium and *Klebsiella pneumoniae*, O-antigen was involved in resistance to serum bactericidal activity (Tomas et al., 1986; Ciurana and Tomas, 1987; Murray et al., 2006). The present study showed that when *A. pleuropneumoniae* strains were cultured in fresh serum, Δ*cpxAR* and Δ*wecA* mutants exhibited lower survival rates than the wild-type strain, while complementation of these strains restored bacterial resistance to serum, which was consistent with previous reports (Tomas et al., 1986; Ciurana and Tomas, 1987; Murray et al., 2006). Based on the above findings, the decreased resistance of the Δ*cpxAR* strain to the bactericidal activity of serum might be caused by lowered *wecA* expression.

Bacterial colonization of the respiratory tract requires the bacteria to overcome a variety of stresses, including oxidative and osmotic stresses, and LPS plays critical roles in this process. To investigate whether the CpxAR TCS and *wecA* were involved in tolerance to oxidative and osmotic stresses, we compared the survival rates of the wild-type, Δ*cpxAR*, CΔ*cpxAR*, Δ*wecA*, and CΔ*wecA* strains when they were exposed to NaCl or H_2_O_2_. The results indicated that the Δ*cpxAR* and Δ*wecA* mutants showed higher sensitivity to oxidative and osmotic stresses than the wild-type and complemented strains. These results suggested that the lack of the Cpx system or O-antigen affected the survival of *A. pleuropneumoniae* under H_2_O_2_ and osmotic stress. Our results are in line with a previous report that an O-antigen mutant exhibited increased sensitivity to H_2_O_2_ and osmotic stress (Berry et al., 2009; Noh et al., 2015; Zheng et al., 2019).

Bacterial burden experimental results showed that the colonization of mutant strains in mouse lung was decreased relative to that of the wild-type, indicating a higher bacterial clearance rate of the Δ*cpxAR* and Δ*wecA* strains. Survival rate assay in a mouse model and histopathological analysis of deceased mice revealed that CpxAR and WecA contributed to *A. pleuropneumoniae* virulence *in vivo*.

To the best of our knowledge, this is the first attempt to demonstrate that CpxAR positively regulates *wecA* expression, which contributes to the growth, stress resistance, and virulence of *A. pleuropneumoniae*. The CpxAR TCS and *wecA* are conserved in many bacteria. Therefore, it would be worthwhile to investigate the relationship between CpxAR and *wecA* in other bacteria. Our findings provide new insight into the pathogenesis of *A. pleuropneumoniae.*

## Data Availability Statement

All datasets generated for this study are included in the article/Supplementary Material.

## Ethics Statement

The animal study was reviewed and approved by Laboratory Animal Monitoring Committee of Huazhong Agricultural University.

## Author Contributions

WB, FY, KY, and HC conceived and designed the experiments. All authors contributed to the acquisition and analysis of the data and the writing of the manuscript.

## Conflict of Interest

The authors declare that the research was conducted in the absence of any commercial or financial relationships that could be construed as a potential conflict of interest.

## Footnotes

1 http://www.softberry.com/berry.phtml?topic=index&group=programs&subgroup=promoter

## Supplementary Material

FIGURE S1 Confirmation of *A. pleuropneumoniae* Δ*wecA* mutant and complemented strain. The Δ*wecA* mutant and complemented strains were verified by PCR with primers weca-exterior-F/R and weca-interior-F/R.

FIGURE S2 Diagram of O-antigen gene cluster in *A. pleuropneumoniae*. **(A)** and co-expression results **(B)**. Lanes 1–4 contain cDNA, RNA without reverse transcription, genomic DNA, and non-template control, respectively.

FIGURE S3 Organization of *wecA* promoter DNA region. The promoter −10 and −35 sites are underlined. The promoter was predicted using Softberry (http://www.softberry.com/berry.phtml?topic=index&group=programs&subgroup=promoter). The CpxR-P binding box is indicated by a gray box.

TABLE S1 Primers used in this study.
