# Supplementary material for: The CpxAR Two-Component System Contributes to Growth, Stress Resistance, and Virulence of Actinobacillus pleuropneumoniae by Upregulating wecA Transcription
Source: Front Microbiol. 2020 May 21;11:1026. doi: 10.3389/fmicb.2020.01026 (PMC7255013; doi:10.3389/fmicb.2020.01026)
Supplement: TABLE S1 — Primers used in this study. [file Table_1.docx]

**Table S1.** Primers used in this study

| **Primers** | Sequence (5′ -3′ ) | | | **Source or reference** | |
| --- | --- | --- | --- | --- | --- |
|  | |  | |  | |
| *hemL*-*wecA*-F/R | | | TGCCCTTGACCTTGGGTGCTA CTTTGTCCGCCGCTGCTA | | This study |
| *wecA*- *APPSER1*_*RS08540*-F/R | | | AATCTTGGGTCGCAATCTGG GTGGCTGGACGAGCAATACA | | This study |
| *APPSER1*_*RS08540*-  *wecB*-F/R | | | AAAGTAGTAGTGGTATCGCCGTGTA CGGTCGTAAAGCGGAGGA | | This study |
| *wecB*-*wecC*-F/R | | | TCGTGTCGGGCGATAGGT GGGAACGGTTCGTCTGGTT | | This study |
| *wecC*-*rffC*-F/R | | | GGAAAGGCTTGCCCGAATA CCCGATCCTGCCGATGTA | | This study |
| *rffC*-*rffA*-F/R | | | CGGCAAACGGACGTAAGACT GCGGCAACGGTTGAAGATA | | This study |
| *rffA* -*wzxE*-F/R | | | TCAAACTCGCCTTTCTCCCT TTCGGATGAACGCAATGACT | | This study |
| *wzxE*- *APPSER1*_*RS08510*-E-F/R | | | GGGTCGGTTCAACTTGGC | | This study |
|  |  |  | TGAGCAGGTCGGCTTATGG | |  |
